# Concrete Crack Monitoring Using a Novel Strain Transfer Model for Distributed Fiber Optics Sensors

**DOI:** 10.3390/s20082220

**Published:** 2020-04-15

**Authors:** Antoine Bassil, Xavier Chapeleau, Dominique Leduc, Odile Abraham

**Affiliations:** 1 COSYS-SII, I4S Team, University Gustave Eiffel, Inria, F-44344 Bouguenais, France; xavier.chapeleau@ifsttar.fr; 2 Quadric, Artelia Group, 14 Porte du Grand Lyon, 01700 Neyron, France; 3 GeM UMR 6183, University of Nantes, F-44322 Nantes, France; dominique.leduc@univ-nantes.fr; 4 GERS-GeoEND, University Gustave Eiffel, IFSTTAR, F-44344 Bouguenais, France; odile.abraham@ifsttar.fr

**Keywords:** strain transfer, imperfect bonding, distributed strains, fiber optics sensors, concrete, crack, crack opening, wedge splitting test

## Abstract

In this paper, we study the strain transfer mechanism between a host material and an optical fiber. A new analytical model handling imperfect bonding between layers is proposed. A general expression of the crack-induced strain transfer from fractured concrete material to optical fiber is established in the case of a multilayer system. This new strain transfer model is examined through performing wedge splitting tests on concrete specimens instrumented with embedded and surface-mounted fiber optic cables. The experimental results showed the validity of the crack-induced strain expression fitted to the distributed strains measured using an Optical Backscattering Reflectometry (OBR) system. As a result, precise estimations of the crack openings next to the optical cable location were achieved, as well as the monitoring of the optical cable response through following the strain lag parameter.

## 1. Introduction

**Figure 1:**
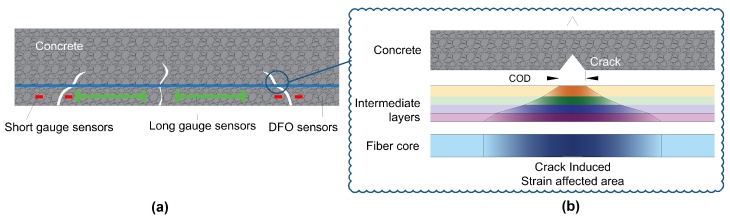
Crack detection using Distributed Fiber Optics Sensing (DFOS) techniques: (**a**) Comparison to traditional sensors, (**b**) strain transferring between layers [1].

Detection and monitoring of crack openings are critical points in the structural health monitoring of civil structures. Early detection and accurate localization of multiple close microcracks with a crack spacing in the order of several centimeters is of interest. In addition, controlling cracks openings within a certain threshold is of paramount importance for reinforced concrete structures, more particularly for structures like nuclear power plants, water reservoirs, and prestressed bridge decks. While several types of sensors and Non Destructive Testing (NDT) techniques can be used for this purpose, optical fibers are particularly interesting because of their ability to perform distributed measurements, making them more likely to intercept cracks propagating in a structural element (Figure 1a). Moreover, the use of thin optical cables is particularly advantageous inside structures where no robust and non-intrusive crack meters or NDT techniques can do the job.

Several devices have already been proposed to detect and localize cracks using Brillouin scattering [2,3,4,5,6] or Rayleigh scattering, in time domain reflectometry [7,8,9,10,11,12] or frequency domain reflectometry [13,14,15,16,17,18]. As a result, a new question arises as to the strain transfer between the host material and the optical fiber, and its potential use for the quantification of crack openings.

Different types of optical cables are available in the market. Among them, some are conceived to be embedded inside the structure during construction, while others are more suitable for surface installation on existing structures. These cables, formed of different materials and shaped in different forms, lead to a different strain transfer response due to shear lag effect in the intermediate layers. In most cases, small information concerning the mechanical properties of the cable and its constituent elements, are available. In addition, the long-term behavior of these cables also remains unknown.

Moreover, localized strain near a propagating crack (Figure 1b) can cause a plastic deformation in intermediate layers or slippage at the interfaces. With the entire complicated phenomenon, monitoring the crack openings quantitatively using an optical cable bonded along a host material is still a challenge and the idea of measuring strain near cracks is always a complicated problem [19]. Recently, methods of estimating crack openings from distributed strain measurements were presented whether by combination to finite element models [15,20] or through calculation of the optical fiber elongation by summing strain gradients [17,21,22]. However, the limitations of these methods prevailed in the cases of multiple crack propagation and until a certain crack opening limit.

Surmounting the mentioned challenges require a good understanding of the mechanical strain transfer near cracks. The case of strain transfer in continuous materials, when no crack exists, was first studied in 1998 by Ansari and Libo [23]. The authors proposed an analytical model for a Silica optical fiber/coating/host material mechanical system. This model is based on the so-called "strain-lag" approach, which was initially developed for the study of bonded assemblies in 1938 by Völkersen [24] and now commonly applied to bonded structural reinforcement composites. In 2006, D. Li et al. [25] proposed an improvement of Ansari’s model for short-length discrete sensors.

Since 2012, three models (analytical and numerical) [5,6,26] were proposed to describe the strain transfer in the presence of a crack in the host material. Imai et al. [5] adapted the assumption proposed previously by Duck et al. [27], by introducing the effect of a crack discontinuity in the host material as a Gaussian distribution at the contact interface with protective coating. It was then used as an input to a finite element model, showing that the crack-induced strain distribution in the optical fiber takes the form of an exponential distribution. Wang et al. [28] assumed that the strain at the crack location is equal to the crack opening over the spatial resolution of the measurement instrument. As this assumption is mainly dependent on the spatial resolution (varying from a measuring instrument to another), it seems to neglect the buffering effect of intermediate layers in the area surrounding the crack location. Therefore, it is considered as a very simplifying approximation affecting the strain transferring theory. Feng et al. [6] introduced the Crack Opening Displacement (COD) in D. Li’s model as an additional local discontinuity in the host material deformation field.

All these models consider a three layer system with perfect bonding between layers. In this paper, we generalize Feng’s approach to a multilayer system with imperfect bonding between layers. The first section is devoted to the presentation of the analytical model. Then, the test set-up and the optical cables are described. The experimental results demonstrate the validity of the model and the precision of the estimated crack openings.

## 2. Strain Transfer Theory for a Multilayer Structure

**Figure 2:**
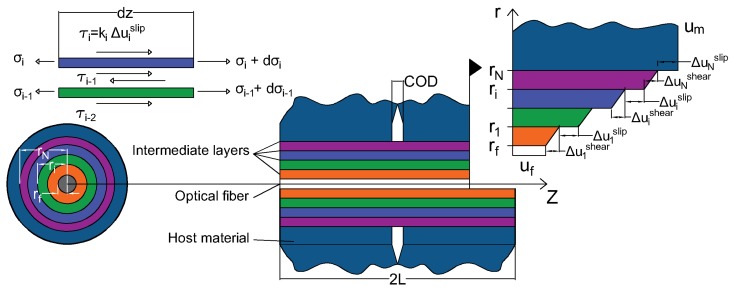
Multiple layers strain transfer system figuring a discontinuity in the host material and an imperfect bonding at different intermediate layers.

The system studied is shown in Figure 2. It consists of N intermediate concentric layers (*i*=1 to N)with the optical fiber at the heart of the assembly. The cylindrical coordinates (r,θ,z) are used, with *z* being the coordinate along the fiber axis.

The coatings deform essentially under the effect of shear stress and the radial displacements $u_{r}^{i}\left(r,z\right)$ are assumed to be small compared to the axial displacements $u_{z}^{i}\left(r,z\right)$. Therefore, the only relevant parameters to describe the system are: $\gamma_{i}\left(r,z\right)$ the engineering shear strain in the $i^{\mathrm{th}}$ layer in the z−r plane, $\tau_{i}\left(r,z\right)$ the shear stress in the z−r plane, $\epsilon_{i}\left(z\right)$ the strain along the *z* direction, and $\sigma_{i}\left(z\right)$ the normal stress in the *z* direction. The other components of the strain and stress tensors can be neglected. Under this assumption, the strain-displacement relationship and the Hook’s law are reduced to:

(1) $$\epsilon_{i}\left(z\right)=\frac{du_{i}\left(z\right)}{dz}≃\frac{\sigma_{i}\left(z\right)}{E_{i}}$$

and

(2) $$\gamma_{i}\left(z,r\right)≃\frac{du_{i}\left(z\right)}{dr}=\frac{\tau_{i}\left(z\right)}{G_{i}}$$

where E and G$=\frac{E}{2\;(1+\nu )}$ are the Young and shear modulus of elasticity, ν being the Poisson’s ratio.

A crack is assumed at the position z=0, which induces an additional discontinuity $\frac{\mathrm{COD}}{2}$. Thus, the displacement $u_{m}\left(r_{N},z\right)$ in the host material in the vicinity of the crack is:

(3) $$\begin{array}{cc} u_{m}\left(r_{N},z\right) & =\int_{0}^{z}\epsilon_{m}\left(z^{\prime }\right)dz^{\prime }+\frac{\mathrm{COD}}{2}\;\mathrm{for}\;z\geq 0 \end{array}$$

where $\epsilon_{m}\left(z^{\prime }\right)$ is the strain in the host material. If this strain is uniform according to *z* or at the most linear:

(4) $$\epsilon_{m}\left(z\right)=K\left(L-|z|\right).$$

As in the case of three-point bending, the displacement becomes equal to:

(5) $$\begin{array}{cc} u_{m}\left(r_{N},z\right) & =K\left(L-\frac{z}{2}\right)z+\frac{\mathrm{COD}}{2}\;\mathrm{for}\;z\geq 0 \end{array}$$

with K representing the strain variation rate along *z*.

The force equilibrium at the interface between two intermediate layers yields:

(6) $$\tau_{i}\left(r,z\right)=\frac{r_{i-1}}{r}\tau_{i-1}\left(r_{i-1},z\right)\;\mathrm{for}\;r_{i-1}\leq r\leq r_{i}.$$

At the interface between the optical fiber and the primary coating, the force equilibrium can be written as:

(7) $$\tau_{f}\left(r_{f},z\right)=-\frac{r_{f}}{2}\;\frac{d\sigma_{f}\left(z\right)}{dz}=\frac{r_{f}E_{f}}{2}\;\frac{d^{2}u_{f}\left(z\right)}{dz^{2}}.$$

In the case of imperfect bonding, the difference in displacement between two consecutive layers has two causes: The shear deformation and the slip at interface. The shear deformation $\Delta u_{i}^{\mathrm{shear}}=u_{i}\left(r_{i},z\right)-u_{i}\left(r_{i-1},z\right)$ can be obtained by integrating Equation (2):

(8) $$\Delta u_{i}^{\mathrm{shear}}=\int_{r_{i-1}}^{r_{i}}\frac{\tau_{i}\left(r,z\right)}{G_{i}}dr.$$

Using Equation (6) leads to:

(9) $$\Delta u_{i}^{\mathrm{shear}}=\frac{\tau_{f}\left(r_{f},z\right)}{G_{i}}\int_{r_{i-1}}^{r_{i}}\left(\frac{r_{i-1}}{r}\right)\left(\frac{r_{i-2}}{r_{i-1}}\right)\ldots \left(\frac{r_{f}}{r_{1}}\right)dr=\frac{\tau_{f}\left(r_{f},z\right)}{G_{i}}\int_{r_{i-1}}^{r_{i}}\frac{r_{f}}{r}dr.$$

Finally, introducing Equation (7) leads to:

(10) $$\Delta u_{i}^{\mathrm{shear}}=-\frac{r_{f}^{2}E_{f}}{2G_{i}}\ln\left(\frac{r_{i}}{r_{i-1}}\right)\frac{d^{2}u_{f}\left(z\right)}{dz^{2}}.$$

Based on the current interfacial state at the coating/host material interface, the slip at the interface between layer *i* and i+1 can be written as:

(11) $$\Delta u_{i}^{\mathrm{slip}}=\frac{\tau_{i}\left(r_{i},z\right)}{k_{i}}$$

where $k_{i}$ is the stiffness parameter depicting the level of interfacial adhesion between two consecutive layers: $k_{i}\to \infty$ corresponds to a perfect bonding state and $k_{i}=0$ to a full debonding state. Using Equation (6), the slip can be rewritten as:

(12) $$\Delta u_{i}^{\mathrm{slip}}=\frac{\tau_{1}\left(r_{1},z\right)}{k_{i}}\left(\frac{r_{i-1}}{r_{i}}\right)\left(\frac{r_{i-2}}{r_{i-1}}\right)\ldots \left(\frac{r_{1}}{r_{2}}\right)=\frac{\tau_{1}\left(r_{1},z\right)}{k_{i}}\left(\frac{r_{1}}{r_{i}}\right).$$

Since $r_{1}\;\tau_{1}\left(r_{1},z\right)=r_{f}\;\tau_{f}\left(r_{f},z\right)$ and using Equation (7), Equation (12) can be written as follows:

(13) $$\Delta u_{i}^{\mathrm{slip}}=-\frac{r_{f}^{2}\;E_{f}}{2r_{i}\;k_{i}}\;\frac{d^{2}u_{f}\left(z\right)}{dz^{2}}.$$

As a result, the multilayer axial displacement compatibility equation is then given by:

(14) $$\sum_{i=1}^{N}\left[\Delta u_{i}^{\mathrm{shear}}+\Delta u_{i}^{\mathrm{slip}}\right]+u_{f}\left(z\right)=u_{m}\left(r_{N},z\right).$$

Introducing Equations (5), (10), and (13) into Equation (14) leads to:

(15) $$-\frac{E_{f}r_{f}^{2}}{2}\left[\frac{1}{G_{1}}\ln\left(\frac{r_{1}}{r_{f}}\right)+\sum_{i=2}^{N}\frac{1}{G_{i}}\ln\left(\frac{r_{i}}{r_{i-1}}\right)+\sum_{i=1}^{N}\frac{1}{r_{i}\;k_{i}}\;\right]\frac{d^{2}u_{f}\left(z\right)}{dz^{2}}+u_{f}\left(z\right)=\left[K\left(L-\frac{z}{2}\right)z+\frac{\mathrm{COD}}{2}\right]$$

and thus:

(16) $$\frac{d^{2}u_{f}\left(z\right)}{dz^{2}}-\lambda^{2}u_{f}\left(z\right)=-\lambda^{2}\left[K\left(L-\frac{z}{2}\right)z+\frac{\mathrm{COD}}{2}\right]$$

where the strain lag parameter λ is equal to:

(17) $$\lambda^{2}=\frac{2}{E_{f}r_{f}^{2}\left[\frac{1}{G_{1}}\ln\left(\frac{r_{1}}{r_{f}}\right)+\sum_{i=2}^{N}\frac{1}{G_{i}}\ln\left(\frac{r_{i}}{r_{i-1}}\right)+\sum_{i=1}^{N}\frac{1}{k_{i}\;r_{i}}\right]}.$$

The general solution of Equation (16) satisfying the boundary conditions: $u_{f}\left(0\right)=0$ and $\epsilon_{f}\left(z\right)\to \pm \epsilon_{m}\left(z\right)$ for z→±∞ is of the form:

(18) $$u_{f}\left(z\right)=\left(\frac{\mathrm{COD}}{2}-\frac{K}{\lambda^{2}}\right)\left(1-e^{-\lambda z}\right)+K\left(L-\frac{z}{2}\right)z.$$

By differentiating Equation (18) with respect to *z*, we obtain the general strain transfer equation for multilayer cable with imperfect bonding:

(19) $$\epsilon_{f}\left(z\right)=\lambda \left(\frac{\mathrm{COD}}{2}-\frac{K}{\lambda^{2}}\right)e^{-\lambda z}+K\left(L-z\right).$$

As the strain varies in concrete structures over important span lengths, the $K/\lambda^{2}$ term is usually lower than 1 μm and therefore can be neglected compared to the crack openings we expect to estimate ($\frac{\mathrm{COD}}{2}>>\frac{K}{\lambda^{2}}$). Thus, a symmetrical strain response measured by the optical fiber around the crack location (at z=0) consists of a crack-induced strain part added to the strain in the host material, whether it is constant:

(20) $$\epsilon_{f}\left(z\right)=\lambda \frac{\mathrm{COD}}{2}e^{-\lambda |z|}+\epsilon_{m}$$

or varies in a linear form:

(21) $$\epsilon_{f}\left(z\right)=\lambda \frac{\mathrm{COD}}{2}e^{-\lambda |z|}+K\left(L-\left|z\right|\right).$$

To sum up, whether a perfect bonding exists at the different interfaces of a multilayer system or a slip occurs with a finite and constant adhesion coefficient, the same exponential form of the crack-induced strain distribution is observed. Moreover, introducing an additional protective or adhesive layer does not affect the strain distribution form when all different layers are behaving in an elastic manner. What differs each case is how the strain lag parameter is related to the characteristics of the system.

## 3. Experimental Campaign

### 3.1. Test Setup

The Wedge Splitting Test (WST) was initially proposed by Linsbauer and Tschegg (in 1986) [29] and developed later by Brühwiler and Wittmann (in 1990) [30] for the study of fracture mechanisms in cementitious materials. The test consists on transforming a vertical force from a stiff steel profile to two splitting forces acting on the specimen.

**Figure 3:**
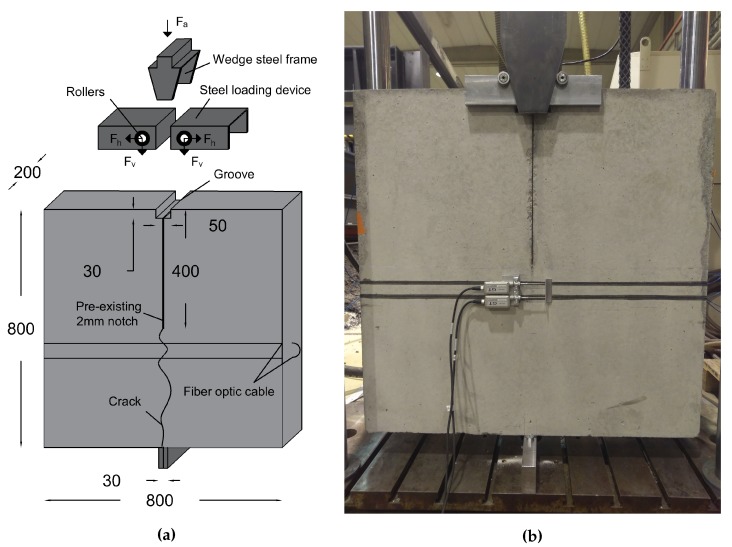
(**a**) Shopdrawing of the testing set-up. (**b**) Front view of the loading arrangement and the concrete specimen instrumented with DFO and LVDT sensors.

Figure 3a shows the geometry of the tested concrete specimens. It is derived from the configuration proposed by Brühwiler [30]. A metallic support with a width equal to 3 cm is located in the center of the specimen and fixed to the lower plate of the testing machine. The main feature of the loading device is the use of wedges and rollers. Two massive steel loading devices equipped with roller bearings on each side are fabricated to be placed on top of the specimen.

Figure 3a shows the geometry of the tested concrete specimens. It is derived from the configuration proposed by Brühwiler [30]. A metallic support with a width equal to 3 cm is located in the center of the specimen and fixed to the lower plate of the testing machine. The main feature of the loading device is the use of wedges and rollers. Two massive steel loading devices equipped with roller bearings on each side are fabricated to be placed on top of the specimen.

The specimens dimensions are 800 mm × 800 mm × 20 mm, with an 8 mm × 5 mm groove and a starter notch of 400 mm. The height of the notch is chosen equal to half the height of the specimen, in order to guarantee a vertical crack propagation [30,31]. The notch is created by fixing a 2 mm thick steel plate in the molds before casting. The surfaces facing the concrete are painted with lubricating oil to reduce the friction between the plate and the concrete during demolding.

Linear Variable Differential Transformer (LVDT) sensors, Linear Variable Differential Transformer covering a 50 mm range, are fixed at the level of the fiber optic lines from both sides of the specimen in order to provide reference measurements of the crack openings. They have a displacement precision of 1 μm and a measurement range of 10 mm. The sensors were calibrated before each test.

The wedge splitting test leads to a vertical propagation of a single tensile crack towards the central metallic support. The height of the specimen allows reach to crack openings of several millimeters before complete failure of the specimen. In addition, a main advantage of the test setup is that the strain in the host material is mainly localized in the fracture process zone during the microcracking stage and it disappears later on during the macrocracking stage. Thus, the strain in concrete can be neglected and evaluating the crack-induced strain becomes more direct using a simplified form of Equation (19):

(22) $$\epsilon_{f}\left(z\right)=\lambda \frac{\mathrm{COD}}{2}e^{-\lambda |z|}$$

In total, 11 specimens equipped with different types of optical cables were tested. The tests were performed after at least 28 days of concrete curing.

### 3.2. The Optical Cables and Their Insertion

**Figure 4:**
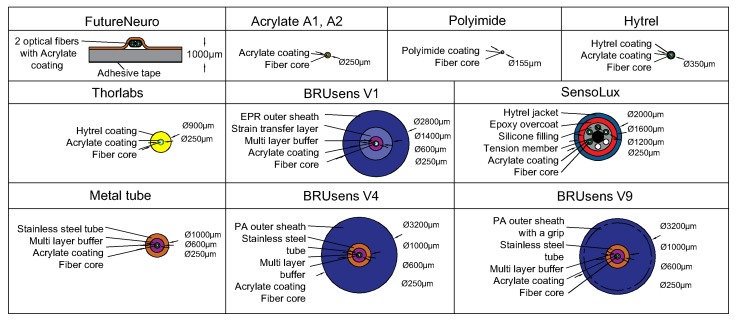
Different types of tested optical cables.

The tested types of cables are listed in Figure 4. Five of them are thin optical fibers (Polyimide, Acrylate A1 and A2, Hytrel, and FutureNeuro), three are robust cables containing a steel layer (Acrylate fiber in Metal tube, BRUsens V4, and V9), and one is a complex non-concentric cable (Sensolux).

**Figure 5:**
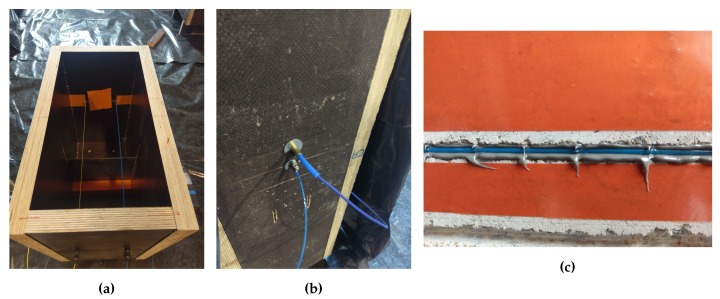
(**a**) View of two fiber optic cables fixed to both sides of the formwork. (**b**) Fiber optic cable fixed to the formwork using dominos. (**c**) Fiber optic cable introduced inside a groove filled with epoxy glue.

These fiber optic cables were whether embedded inside the specimen or glued in a U-groove engraved on the surface. In order to make sure that the cables keep their same position, they were strained (Figure 5a) and fixed to the two sides of the formwork using dominos (Figure 5b).

After removing the formwork, the U-groove was sewed perpendicularly to the estimated vertical direction of the crack. The dimensions of the groove were adapted to the diameter of each type of fiber optic cable. After adding a first layer of epoxy glue, the cable is introduced inside (Figure 5c) and then covered with a second layer to fill the groove.

**Figure 6:**
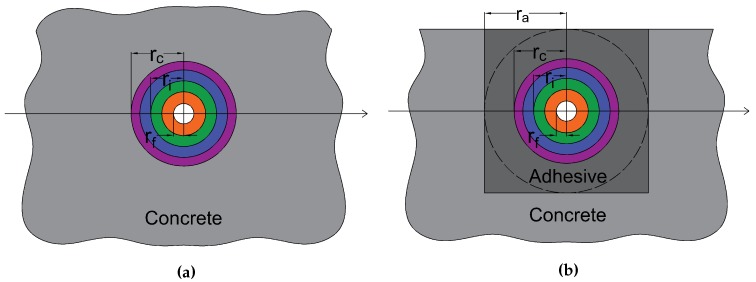
(**a**) Embedded cable; (**b**) Surface-mounted cable.

For embedded cables, the geometry of insertion corresponds exactly to the assumptions of the model (see Figure 6a).

Then, according to Equation (17), the strain lag parameter can be expressed as:

(23) $$\lambda_{\mathrm{emb}}^{2}=\frac{2}{E_{f}r_{f}^{2}\left[\frac{1}{G_{1}}\ln\left(\frac{r_{1}}{r_{f}}\right)+\sum_{i=2}^{N}\frac{1}{G_{i}}\ln\left(\frac{r_{i}}{r_{i-1}}\right)+\sum_{i=1}^{N-1}\frac{1}{k_{i}r_{i}}\right]+\frac{E_{f}r_{f}^{2}}{k_{m/c}r_{c}}}$$

where $k_{m/c}$ is the interfacial adhesion coefficient at the host material/cable interface. For surface-mounted cables, the modelization is not so straightforward. Given the dimension of the groove, the epoxy glue can be considered as an additional concentric layer of radius $r_{a}$, surrounded by the host material as illustrated in Figure 6b. The strain lag parameter is then given by:

(24) $$\lambda_{\mathrm{surf}}^{2}=\frac{2}{E_{f}r_{f}^{2}\left[\frac{1}{G_{1}}\ln\left(\frac{r_{1}}{r_{f}}\right)+\sum_{i=2}^{N}\frac{1}{G_{i}}\ln\left(\frac{r_{i}}{r_{i-1}}\right)+\sum_{i=1}^{N-1}\frac{1}{k_{i}r_{i}}\right]+\frac{E_{f}r_{f}^{2}}{k_{a/c}r_{c}}+\frac{E_{f}r_{f}^{2}}{k_{m/a}r_{a}}+\frac{E_{f}r_{f}^{2}}{G_{a}}\ln\left(\frac{r_{a}}{r_{c}}\right)}$$

where $k_{a/c}$ and $k_{m/a}$ are the interfacial adhesion coefficients at the adhesive/cable and host material/adhesive interfaces and G${}_{a}$ the shear modulus elasticity of the adhesive.

**Figure 7:**
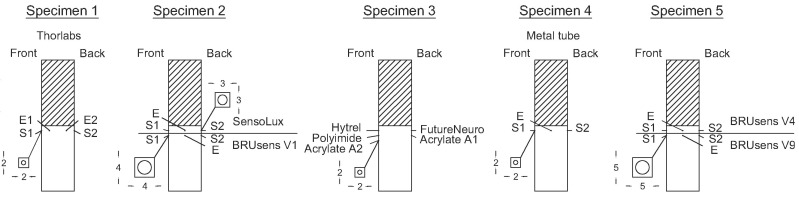
Shop drawing of the specimen’s fiber-optic instrumentation policy.

The five concrete specimens are presented in Figure 7, where "E" and "S" correspond to Embedded and Surface-mounted lines respectively. Figure 7 also presents the groove dimensions corresponding to each type of optical cable.

During the tests, distributed strain measurements were performed using the ODISI–B interrogation unit (manufactured by Luna). Each wedge splitting test was controlled under a vertical displacement rate of 0.2 mm/min and performed until the crack opening at all different optical fiber lines reached at least 2 mm. The ODISI-B interrogator was continuously measuring at a frequency equal to 20 Hz using the high resolution operating mode (spatial resolution of 1.3 mm).

## 4. Validation of the Mechanical Strain Transfer Model

The objective of this section is to show that Equation (22) covers different types of cables in different insertion configurations on the structure. Firstly, two parameters are examined: Maximum amplitude (peak value) and the shape of the strain profiles measured by the fiber.

**Figure 8:**
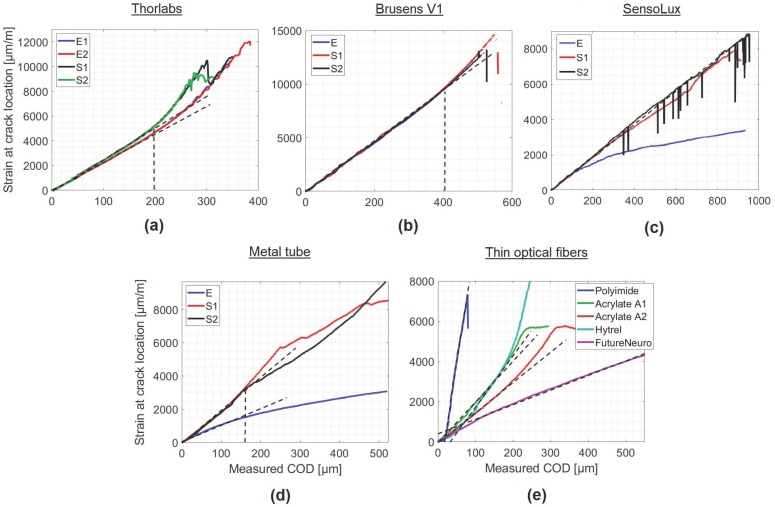
Peak strain variation $\epsilon_{f}^{\max}$ at the crack location for the different optical fibers in function of the measured crack opening: (**a**) Thorlabs, (**b**) BRUsens V1, (**c**) SensoLux, (**d**) Metal tube, (**e**) Thin optical fibers.

The maximum amplitude of the strain measured by the fiber $\epsilon_{f}^{\max}$ is the strain at the center of the crack. This peak strain value should be equal to $\epsilon_{f}\left(0\right)=\lambda \frac{\mathrm{COD}}{2}$ according to Equation (22). Therefore, a linear variation of $\epsilon_{f}^{\max}$ in function of the crack opening displacement is expected, as long as Equation (22) holds true. Figure 8 shows the variation of the maximum strain amplitude as a function of the crack opening measured by LVDT sensors.

Nine of the eleven tested optical fibers figured the same behavior where $\epsilon_{f}^{\max}$ varies linearly with the crack opening until a threshold, ${\mathrm{COD}}_{\max}$, where it deviates from linearity. This threshold separates the region where the cable has an elastic behavior from the region where it has a post-plastic nonlinear behavior. Its value depends critically on the used cable. It varies from 80 μm for the Polyimide fiber to 1400 μm for the FutureNeuro cable (Figure 8e), which was glued directly to the surface of the concrete specimen. For most of the optical cables with an external diameter less than 1 mm (Figure 8a,d,e), COD${}_{\max}$ is comprised between 150 μm and 200 μm. Thicker optical cables, the SensoLux and BRUsens V1 cables (Figure 8b,c), figured higher ${\mathrm{COD}}_{\max}$ of respectively 400 μm and 900 μm. It is important to mention that some optical cables, i.e. the Metal tube and SensoLux cables (Figure 8c,d), figured lower ${\mathrm{COD}}_{\max}$ levels when embedded inside the concrete material compared to the glued on the surface. This could be due to an early unstable cable/concrete adherence in the absence of the epoxy adhesive layer. The extremely smooth surface of the SensoLux and Metal tube cables could be the main reason behind this change in behavior. Smooth surface is usually an important factor in increasing the adherence with epoxy adhesives. On the other hand, rugged surface can be more convenient for a good bonding with non-homogeneous materials like concrete.

**Figure 9:**
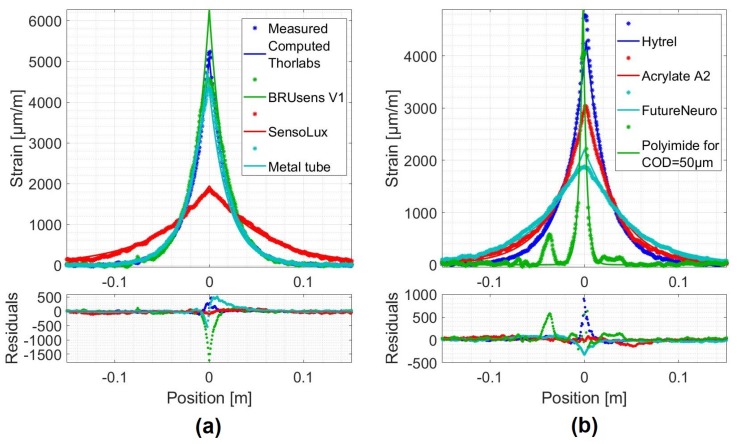
Measured crack-induced strain distribution at a Crack Opening Displacement (COD) = 200 μm compared to the one computed using the analytical model for different optical cables: (**a**) Thorlabs, BRUsens V1, SensoLux and Metal tube; (**b**) Hytrel, Acrylate A2, FutureNeuro and Polyimide fibers.

As a second step, and once the crack opening validity range has been established, the shape of the measured strain distribution profiles should conform to the model depicted by Equation (22). Figure 9 presents the strain profiles measured over the different cables when the crack opening is equal or close to COD${}_{\max}$ (stars). In addition, the associated computed fits using Equation (21) are also presented (solid lines).

A crack opening of 200 μm was chosen, where the majority of the tested optical cables behave in an elastic manner. Except for the Polyimide fiber, where the measured and computed strain distributions corresponding to a COD = 50 μm were plotted (Figure 9).

Figure 9 also shows the residuals, which are the differences between the measurements and the fits. A good agreement between measured and computed distributions is observed. Indeed, the residuals never exceeded 20% of the measured strain. As the Polyimide fiber’s strain distribution profiles covered a relatively small part of the fiber, increased residuals at ±25 mm are observed (Figure 9b). These residuals were not due to a weakness in the model but were most likely due to the aluminum pieces glued at these positions that held the LVDT sensor. The exponential shape predicted by the model is then confirmed. This forms a second argument of the relevance of the proposed analytical model.

**Figure 10:**
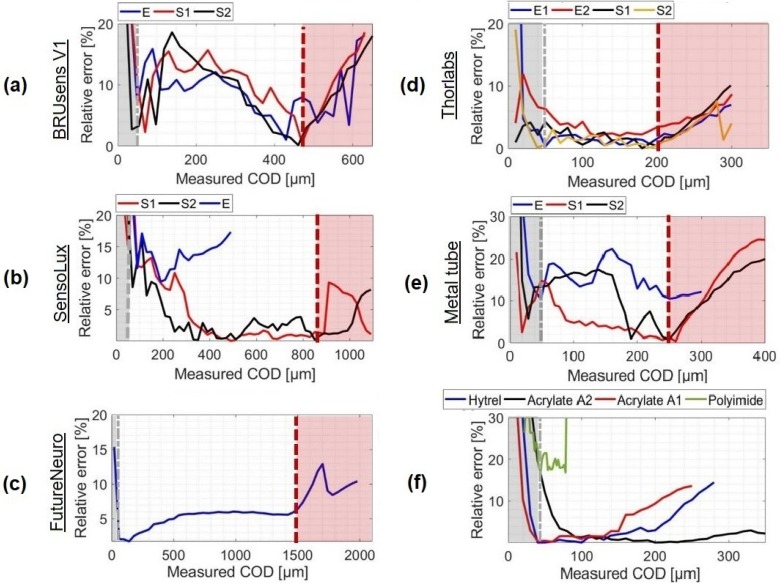
Estimated COD for different optical sensor configurations compared to those measured by the LVDT sensors: (**a**) BRUsens V1, (**b**) SensoLux, (**c**) FutureNeuro, (**d**) Thorlabs, (**e**) Metal tube, (**f**) Thin Optical fibers.

It then remains to prove the accuracy of the crack opening displacements deduced from the fits. Figure 10 shows the relative difference between the crack openings measured with the optical fiber and LVDT sensors.

The LVDT sensors are used as reference sensors and therefore this difference can be considered as an estimation error from the optical fiber measurement.

It can be seen that the error did not exceed the 20% in the COD range $\left[50\;\mu m;{\mathrm{COD}}_{\max}\right]$ for all the cables and most varies around 2–5%. These values are consistent with the variability often observed with concrete and prove the validity of the measured CODs.

Exceeding the ${\mathrm{COD}}_{\max}$, the relative error starts increasing progressively proving that the model is no longer valid. Some types of optical cables (Figure 10b,c) figured a slower transition phase where residuals increased at a lower rate and stayed localized around the crack location. For these types of cables, i.e. the SensoLux and FutureNeuro cables, the relative error did not exceed 15% in the post-elastic range.

For crack openings of less than 50 μm, the high increase in the relative errors were most likely due to the limits in terms of strain accuracy at the level of the measurement system or the host material itself. At the system level, the distributed strain measurements were characterized by a strain repeatability of only ±20 μm/m. At the material level, multiple micro discontinuities are usually formed with residual strains in the fracture process zone before the macro crack starts propagating. Thus, assuming the presence of a crack surrounded by negligible strains in concrete is not totally true in the microcracking stage. Despite these possible sources of errors, some optical fibers, like Thorlabs, were able to reach a relative error of less than 10% at a crack opening of only 10 μm. Higher accuracy cannot be evaluated using the reference LVDT sensor as it is limited to a precision of 1 μm.

**Figure 11:**
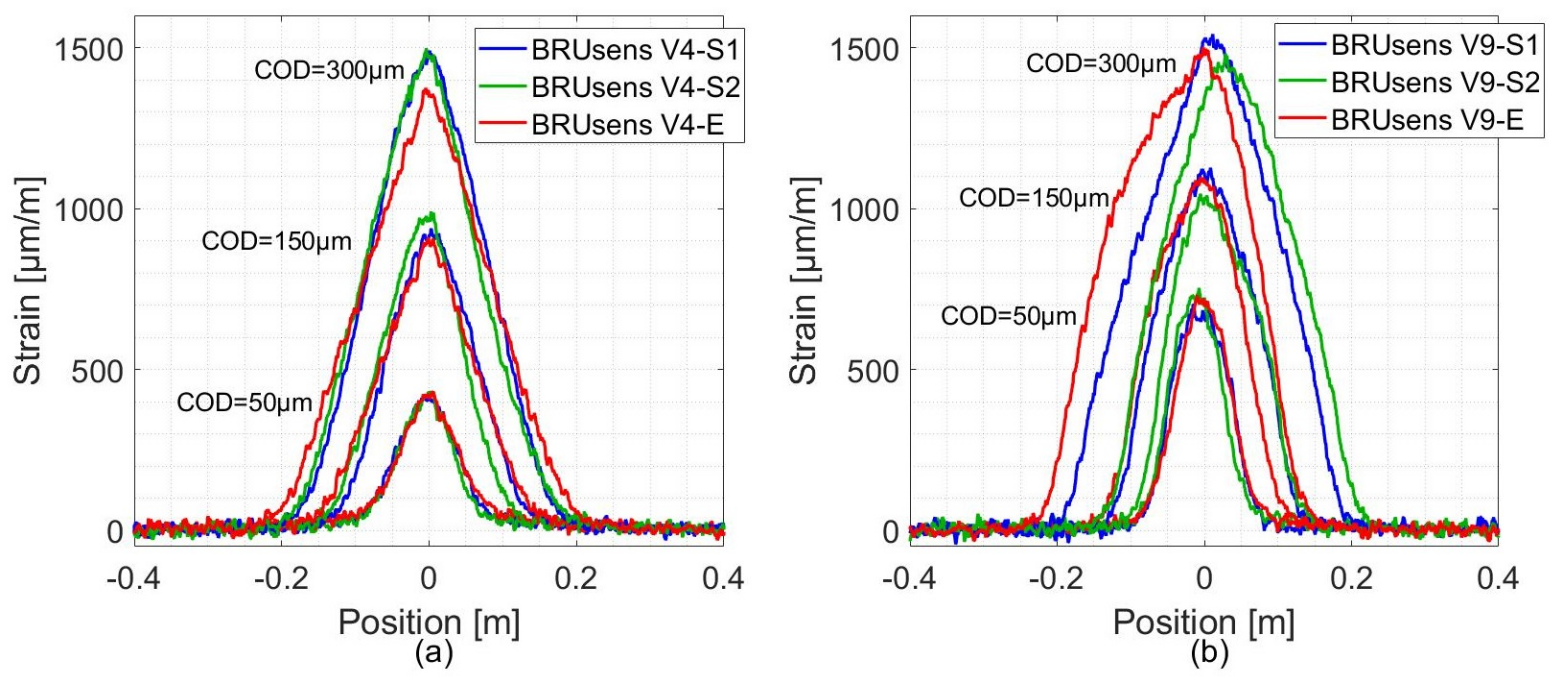
The measured strain spatial distribution under different crack openings from: (**a**) BRUsens V4 and (**b**) V9 fiber optic lines.

As for the robust optical cables, Figure 11 shows the measured strain profiles for BRUsens V4 and V9 cables. These cables figure an additional PolyAmide (PA) outer sheath surrounding the Metal tube. The crack-induced strain distribution takes a triangular form under different crack openings. The corresponding fitted curves are not plotted in Figure 11 as they lead to high residual levels and high error in COD estimations. In the lead up to the mechanical strain transfer equation, and in order to establish Equation (7), the Young modulus of the intermediate layers were neglected compared to the Young modulus of the optical fiber (72 GPa). The fact that this assumption is not true in the case of robust cables could be the reason behind their particular response, as they figure a stainless steel layer characterized by a high Young modulus (200 GPa). Given the repeatability and consistency of the simple triangular shape obtained from the strain profiles of BRUsens V4 and V9 cables, it is later possible to propose a more adapted analytical model.

## 5. Strain Lag Parameter Evaluation

**Figure 12:**
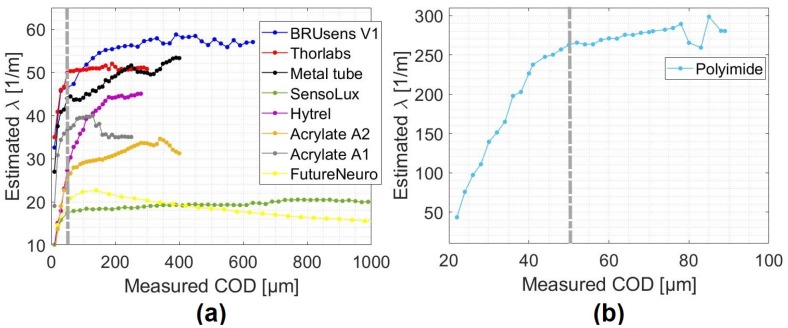
Variation of the strain lag parameter λ as a function of the measured COD for (**a**) all the tested cables mounted on the surface of the specimens and for the (**b**) Polyimide fiber.

As the strain lag parameter holds the different geometrical and mechanical properties of the multilayer strain transfer system, monitoring its variations can help in understanding the response of the system. Figure 12 shows the estimated strain lag parameter values from the fitting process for different cables mounted on the surface of the specimens.

As expected from Equation (24), the strain lag parameter varied from one cable to another since it depends on the constitution of the cable and the adherence between each two consecutive layers. However, the same behavior is observed for all the cables: The strain lag parameter increased during the microcracking stage and then stabilized until it reached an asymptotic value, which can be considered as the real strain lag value for the cable. The strain lag parameters are comprised between 20 m${}^{-1}$ and 60 m${}^{-1}$ (Figure 12a) except for the Polyimide cable where it varies around 260 m${}^{-1}$ (Figure 12b). The measured strain was not only proportional to COD but also to λ. This means that the Polyimide fiber figures crack-induced strain distributions was 5 to 15 times higher than the other tested cables, which allowed for the accurate detection of tiny crack propagation. Associating this fiber to a DFOS system with improved strain precision could lead to the detection of submicron cracks.

**Figure 13:**
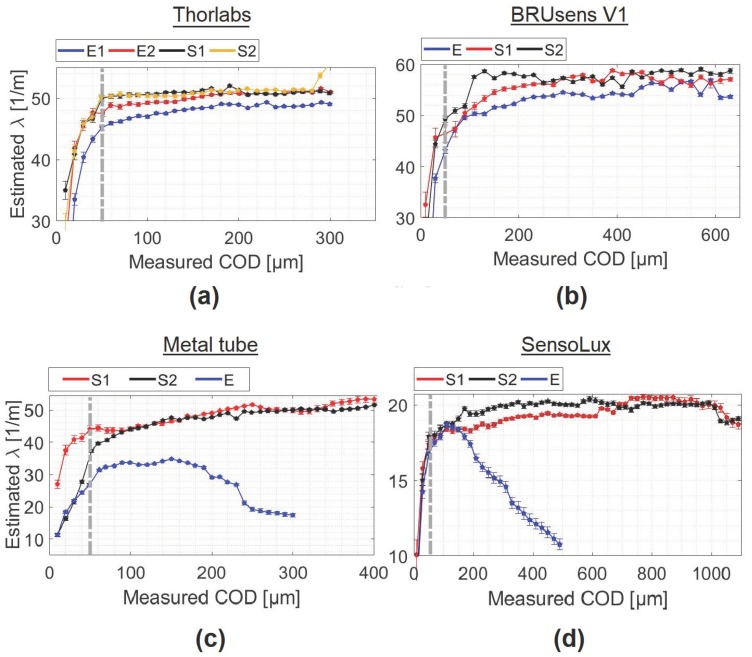
Comparison of the strain lag parameter λ for embedded and surface-mounted lines of (**a**) Thorlabs, (**b**) BRUsens V1, (**c**) SensoLux, and (**d**) Metal tube.

Figure 13 shows the strain lag parameter given by the fit for embedded Thorlabs, BRUsens V1, Metal tube, and SensoLux cable.

On the same figure, the strain lag parameter values for surface lines are also plotted for comparison. In all cases, the strain lag parameter values for embedded cables are lower than those for surface-mounted cables.

According to Equations (23) and (24), the difference in strain lag parameter values for an embedded and a surface mounted cable can be expressed as:

(25) $$\frac{1}{\lambda_{\mathrm{emb}}^{2}}-\frac{1}{\lambda_{\mathrm{surf}}^{2}}=\frac{E_{f}r_{f}^{2}}{2}\left[\frac{1}{k_{m/c}r_{c}}-\left(\frac{1}{k_{m/a}r_{a}}+\frac{1}{k_{a/c}r_{c}}+\frac{1}{G_{a}}\ln\frac{r_{a}}{r_{c}}\right)\right]$$

Assuming a perfect bonding between the different layers, this difference reduces to: $-\frac{E_{f}r_{f}^{2}}{2G_{a}}\ln\frac{r_{a}}{r_{c}}$. As the latter can only have negative values, this means that the strain lag parameter of the embedded cable is expected to be greater than the strain lag parameter of the surface mounted cable for perfect bonding. Based on the tests results, the observed difference between the two insertion methods must then be attributed to a case with an imperfect bonding between layers. Therefore, based on Equation (17), $\lambda_{\mathrm{emb}}<\lambda_{\mathrm{surf}}$ implies: $\frac{1}{k_{m/c}r_{c}}>\frac{1}{k_{m/a}r_{a}}+\frac{1}{k_{a/c}r_{c}}$. As a result, we can conclude that the epoxy adhesive layer provides higher interfacial adhesion with the cable and the concrete material compared to the one existing when the concrete and the cable are directly in contact. Moreover, in the case of Metal tube and SensoLux cable (Figure 13c,d), this additional layer helped stabilizing the cable/concrete interfacial adherence over a broader crack opening range.

The characteristics of the cables determined from wedge splitting tests are listed in Table 1.

The larger the strain lag parameter, the greater the sensitivity to crack propagation, but the smaller the ${\mathrm{COD}}_{\max}$. The resolution and the dynamic range ($\left[{\mathrm{COD}}_{\min};{\mathrm{COD}}_{\max}\right]$) of the cable thus depend directly on the strain lag parameter. It appears that the strain lag parameter could be used as a control parameter of the fit when its asymptotic value is known. Indeed, the best accuracy was obtained when the strain lag was close to its asymptotic value. When it was significantly lower, the error increased as deduced from the Metal tube and SensoLux embedded lines. This is due to the fact that a lower value reflects the loss in adhesion between layers. Through more experimental tests, an empirical relationship between the decrease in the strain lag parameter and the increase in the relative COD estimation error can be established.

It is important to mention that additional wedge splitting tests were performed in [32] showing that concrete hardening, epoxy glue aging, and bonding lengths can affect the strain lag parameter values. These results put the spotlight on the importance of continuously estimating λ values during the life of a structure and thus, the advantage of the established methodology compared to those presented in the literature that assume a constant λ value for each cable.

## 6. Conclusions

We proposed a strain transfer model for multilayer systems that takes into account imperfect interfacial bonding between layers. This analytical model includes the slip or discontinuities at the interfaces by introducing an interfacial stiffness parameter. The key result of our calculations is that the crack-induced strain distribution can take the form of:

(26) $$\epsilon_{crack}\left(z\right)=\frac{\mathrm{COD}}{2}\lambda e^{-\lambda \;|z|}$$

which only depends on two parameters: COD and λ. The parameter COD represents the opening of the crack. The parameter λ, or strain lag parameter, is related to the characteristics of the studied system and depends on the mechanical properties of the different layers as well as the adhesion at common interfaces. Therefore, its evolution provides information on the evolution of the optical cable/host material system under study.

Having a single law of behavior covering a wide range of configurations is of undeniable interest from an experimental point of view. Indeed, in many practical cases, analyzing the strain measurements only consists on fitting the mechanical strain transfer function composed of the crack-induced strain part (Equation (26)) and the elastic strain in the host material. If the fit is relevant, the crack opening is obtained directly, without precise knowledge of the internal composition of the system and the interactions between layers, and without the need to perform prior calibration tests.

In order to validate this model, wedge splitting tests were performed on specimens instrumented with optical fiber cables on the surface and inside the concrete material, together with LVDT extensometers, which serve as reference sensors. The spatial strain distribution was measured using a Rayleigh based interrogation unit and fitted using Equation (26) to estimate the crack opening and strain lag parameter. Crack openings were compared to reference measurements, showing low relative errors of 2% for some optical fibers and up to maximum crack opening of 200–1500 μm.

Optical cables embedded inside the concrete material showed less accurate results with relative errors of 10%. Through monitoring the strain lag parameter variation, we were able to associate this discrepancy in crack opening estimation accuracy to different levels of bonding at the interfaces. The increased interfacial adhesion between the added adhesive layer and both the optical cable and concrete material increases the strain lag coefficient and in some cases guarantees the steadiness and stability of the interfacial bonding.

These results show that the distributed optical fiber strain sensing associated with the law of behavior (Equation (26)) is an effective method for monitoring the opening of cracks in concrete structures. It also allows the examination and monitoring of the condition of the optical cable inside the concrete material throughout the life of the structure.

As a next step, the durability of the system should be examined. The behavior of the optical cable under mechanical and environmental cyclic loading should be studied. The possibility of employing optical cables with different levels of shear lag parameter and therefore different levels of sensitivity to microcracks can be useful for investigating more complex cracking behaviors in novel materials like ultra-high-performance fiber reinforced concrete (UHPFRC).

## Figures and Tables

**Table 1:** Characteristics of the cables.

	Strain Lag	Dynamic Range
Polyimide	260 m${}^{-1}$	[50 μm; 80 μm]
Acrylate A1	32 m${}^{-1}$	[50 μm; 200 μm]
Acrylate A2	34 m${}^{-1}$	[50 μm; 200 μm]
Hytrel	44 m${}^{-1}$	[50 μm; 200 μm]
Metal tube	48 m${}^{-1}$	[50 μm; 160 μm]
Thorlabs	50 m${}^{-1}$	[50 μm; 200 μm]
BRUsens V1	58 m${}^{-1}$	[50 μm; 400 μm]
SensoLux	20 m${}^{-1}$	[50 μm; 900 μm]
FutureNeuro	15 m${}^{-1}$	[50 μm; 1500 μm]
